# Overview and new records of the species of the tribes Dyschiriini and Clivinini from Iraq (Coleoptera, Carabidae, Scaritinae)

**DOI:** 10.3897/zookeys.672.11885

**Published:** 2017-05-09

**Authors:** Petr Bulirsch, Mieczysław Stachowiak

**Affiliations:** 1 Milánská 461, CZ-109 00 Praha 111, Czech Republic; 2 UTP University of Science and Technology; ul. Sucha 9, 85-796 Bydgoszcz, Poland

**Keywords:** Carabidae, *Clivina*, Clivinini, *Clivinopsis*, Coleoptera, Dyschiriini, *Dyschiriodes*, *Dyschirius*, Iraq, key, Scaritinae, *Torretassoa*

## Abstract

The genera *Clivinopsis* Bedel 1895 and *Torretassoa* Schatzmayr & Koch 1933 have been recorded in Iraq for the first time. New records of several species of *Dyschirius* Bonelli 1810 *Dyschiriodes* Jeannel 1941 (Dyschiriini W. Kolbe 1880) and *Clivina* Latreille 1802 (Clivinini Rafinesque 1815) are given. The identification key to the species of Dyschiriini from Iraq is provided.

## Introduction

The scaritine tribes Clivinini Rafinesque, 1815 and Dyschiriini W. Kolbe, 1880 are distributed almost worldwide. The former includes several genera and several hundred species, and the latter includes several genera and few hundred species. The knowledge about the Iraqi species of the two tribes is incomplete. The species were reported from Iraq, without exact localities provided (Ali 1966, Balkenohl 2003). Fedorenko (1996) listed exact localities of some dyschiriine species, while only mapped them for some others.

## Material and methods

This article is based on the collections listed below. The specimens collected by Z. Stebnicka and J. Pawłowski during the 1977–1978 expedition of ISEA to Iraq were identified by the second author and the remaining specimens by the first author (mostly placed in his collection). The acronyms used are as follows:

**ISEA** The Institute of Systematics and Evolution of Animals of the Polish Academy of Sciences (Instytut Systematyki i Ewolucji Zwierząt Polskiej Akademii Nauk), Kraków, Poland;

**HNHM** Hungarian Natural History Museum, Budapest, Hungary (Magyar Természettudományi Múzeum);

**PBPC** collection of Petr Bulirsch, Prague, Czech Republic.

Abbreviations used in the key are as follows:

**B** basal setiferous puncture;

**D** dorsal setiferous punctures;

**A** apical setiferous punctures;

**H** (post)humeral setiferous punctures,

**SP** setiferous punctures;

**L** length of body.

## Results

### Tribe *Clivinini* Rafinesque, 1815

#### Genus Clivina

Taxon classificationAnimaliaColeopteraCarabidae

Latreille, 1802

##### Note.

This genus comprises approximately 650 species distributed almost worldwide.

#### Clivina (Clivina) ypsilon

Taxon classificationAnimaliaColeopteraCarabidae

Dejean, 1830

Clivina (Clivina) ypsilon Dejean, 1830; Ali 1966: 15.

##### New records.

1 specimen: Hatra, 3 v 1978, at light in a hotel, leg. Z. Stebnicka, (ISEA); 1 specimen: S. E. Iraq, Amara, vi.1956, K. Khalaf coll., (PBPC).

##### Comments.

A widespread, common and variable species reported from exact localities in Iraq for the first time.

#### Clivina (Clivina) euphratica

Taxon classificationAnimaliaColeopteraCarabidae

Putzeys, 1866

Clivina (Clivina) euphratica Putzeys, 1866; Ali 1966: 15; Balkenohl 2003: 219.

##### Comment.

A poorly known species described from Iraq.

#### Clivina (Leucocara) laevifrons

Taxon classificationAnimaliaColeopteraCarabidae

Chaudoir, 1842

Clivina (Leucocara) laevifrons Chaudoir, 1842; Ali 1966: 15; Balkenohl 2003: 220.

##### Comment.

A common species widespread in the Mediterranean area to the Middle Asia. Ali (1966) properly keyed its characters, but by mistake omitted the species name in the key.

### Tribe *Dyschiriini* W. Kolbe, 1880

#### Genus Dyschirius

Taxon classificationAnimaliaColeopteraCarabidae

Bonelli, 1810

##### Note.

This genus in the sense of Fedorenko (1996) comprises nearly 20 mostly Palearctic taxa; one of them is known also from Iraq.

#### Dyschirius beludscha ganglbaueri

Taxon classificationAnimaliaColeopteraCarabidae

Znojko 1927

Dyschirius beludscha ganglbaueri Znojko 1927; Fedorenko 1996: 80: (Iraq, Baghdad, Abu-Ghraib, v.1984, Ing. Smatana).

##### New record.

1 specimen: Iraq, Kirkuk, lgt. W. Schors, (PBPC).

##### Comment.

A common subspecies widespread in NW Africa to the Middle Asia.

#### Genus Dyschiriodes

Taxon classificationAnimaliaColeopteraCarabidae

Jeannel, 1941

##### Note.

Unlike Balkenohl (2003), Fedorenko (1996) considers this taxon as independent genus, not subgenus of *Dyschirius*. It is very largely distributed and includes five subgenera with over 300 species and subspecies, including ten hitherto reported from Iraq.

#### Dyschiriodes (Dyschiriodes) agnatus

Taxon classificationAnimaliaColeopteraCarabidae

(Motschulsky, 1844)

Dyschiriodes (Dyschiriodes) agnatus (Motschulsky, 1844); Ali 1966: 15 (as D. lucidus Putzeys, 1866); Fedorenko 1996: 157 (geographical distribution map); Balkenohl 2003: 224.

##### New record.

1 specimen: Iraq, Tekrit, ii-v.1979, (PBPC).

##### Comment.

A very common species widespread from NW Africa to W Kazakhstan.

#### Dyschiriodes (Dyschiriodes) auriculatus

Taxon classificationAnimaliaColeopteraCarabidae

(Wollaston, 1867)

Dyschiriodes (Dyschiriodes) auriculatus (Wollaston, 1867); Fedorenko 1996: 173 (Tekrit); Balkenohl 2003: 224.

##### New record.

5 specimens: Iraq, Tekrit, ii-v.1979, (PBPC).

##### Comment.

A rather common species widespread from NW Africa to the Middle Asia.

#### Dyschiriodes (Dyschiriodes) cariniceps

Taxon classificationAnimaliaColeopteraCarabidae

(Baudi di Selve, 1864)

Dyschiriodes (Dyschiriodes) cariniceps (Baudi di Selve, 1864); Mařan 1935: 211 (Dyschirius kalalae; Baghdad); Fedorenko 1996: 185; Balkenohl 2003: 225.

##### New records.

1 specimen (ab. kalalae): Iraq, Mosul, 28.vii.1956; 2 specimens (non-aberrant): Iraq, 80 km SW Baghdad, Shitatha oasis, creek bed, (PBPC).

##### Comment.

A rather common species populating Sardinia, Sicilia, North Africa and extending eastward as far as Iraq and Iran.

#### Dyschiriodes (Dyschiriodes) clypeatus

Taxon classificationAnimaliaColeopteraCarabidae

(Putzeys, 1866)

Dyschiriodes (Dyschiriodes) clypeatus (Putzeys, 1866); Ali 1966: 16; Fedorenko 1996: 189 (as D. clypeatus perlongus (Müller, 1937); Baghdad); Balkenohl 2003: 225.

#### Dyschiriodes (Dyschiriodes) euphraticus

Taxon classificationAnimaliaColeopteraCarabidae

(Putzeys, 1846)

Dyschiriodes (Dyschiriodes) euphraticus (Putzeys, 1846); Ali 1966: 16 (also as D. tuberculifer Müller, 1922); Fedorenko 1996: 176 (‘Mesopotamien’ and ‘Euphrates’); Balkenohl 2003: 225.

##### Comment.

Not a frequent species in Turkey, Iraq and Iran.

#### Dyschiriodes (Dyschiriodes) jedlickai

Taxon classificationAnimaliaColeopteraCarabidae

(Kult, 1940)

Dyschiriodes (Dyschiriodes) jedlickai (Kult, 1940); Kult 1946: 1 (Mesopotamie, Euphrat); Fedorenko 1996: 157 (as D. agnatus, part.); Balkenohl 2003: 226 (as D. agnatus, part.); Bulirsch and Fedorenko 2007: 5.

##### Revised material.

1 specimen: Iraq, Euphrat, (PBPC).

##### Comment.

A rare species in Turkey and Iraq (one old record).

#### Dyschiriodes (Dyschiriodes) mesopotamicus

Taxon classificationAnimaliaColeopteraCarabidae

(Müller, 1922)

Dyschiriodes (Dyschiriodes) mesopotamicus (Müller, 1922); Ali 1966: 16; Fedorenko 1996: 184 (geographical distribution map); Balkenohl 2003: 226.

##### Comment.

Not a common species, distributed from Turkey to Middle Asia.

#### Dyschiriodes (Dyschiriodes) pusilluspusillus

Taxon classificationAnimaliaColeopteraCarabidae

(Dejean, 1825)

##### New records.

1 specimen: Iraq, Tekrit, ii-v.1979, (PBPC); 15 specimens: Iraq: Tharthar Lake, vii–viii. 1977, leg. J. Pawłowski, 21 specimens: 15 km W Kerbala, 14.v.1978, leg. Z. Stebnicka, (ISEA).

##### Comment.

Some specimens of *D. pusillus pusillus* are very similar to those of *D. clypeatus* and its differentiation is difficult. It is recorded in Iraq for the first time.

#### Dyschiriodes (Dyschiriodes) salinusstriatopunctatus

Taxon classificationAnimaliaColeopteraCarabidae

(Putzeys, 1846)

Dyschiriodes (Dyschiriodes) salinus striatopunctatus (Putzeys, 1846); Balkenohl 2003: 227.

##### New record.

1 specimen: Iraq: Razeza Lake, 15 km W Kerbala, 14 v 1978, leg. Z. Stebnicka, (ISEA).

##### Comment.

A very common subspecies, very largely distributed from CE Europe, N Africa to Mongolia.

#### Dyschiriodes (Eudyschirius) importunusimportunus

Taxon classificationAnimaliaColeopteraCarabidae

(Putzeys, 1857)

Dyschiriodes (Eudyschirius) importunus importunus (Putzeys, 1857); Fedorenko 1996: 128 (geographical distribution map); Balkenohl 2003: 229

##### New records.

1 specimen: Iraq: Hatra, 3.v.1978, leg. Z. Stebnicka, (ISEA); 1 specimen: Iraq, Baguba [= Baqubah], (PBPC).

##### Comment.

A common subspecies distributed from Italy to Mesopotamia.

### *Genus Torretassoa* Schatzmayr & Koch, 1933

#### Torretassoa alfierii

Taxon classificationAnimaliaColeopteraCarabidae

Schatzmayr & Koch, 1933

##### Studied material.

Paratypus, Egitto, Helwan, 18.ii.1935, W. Wittmer; 5 specimens: Iran Prov. Markazi, Kavir Desert, Houz-e Soltan, 830m, 3 km S Kushk-e Nosrat; 35°5'14"N, 50°55'26"E, at light, 28.vi.2000, leg. Kálmán Székely, (HNHM, PBPC);

##### New record.

**Figure 1.**
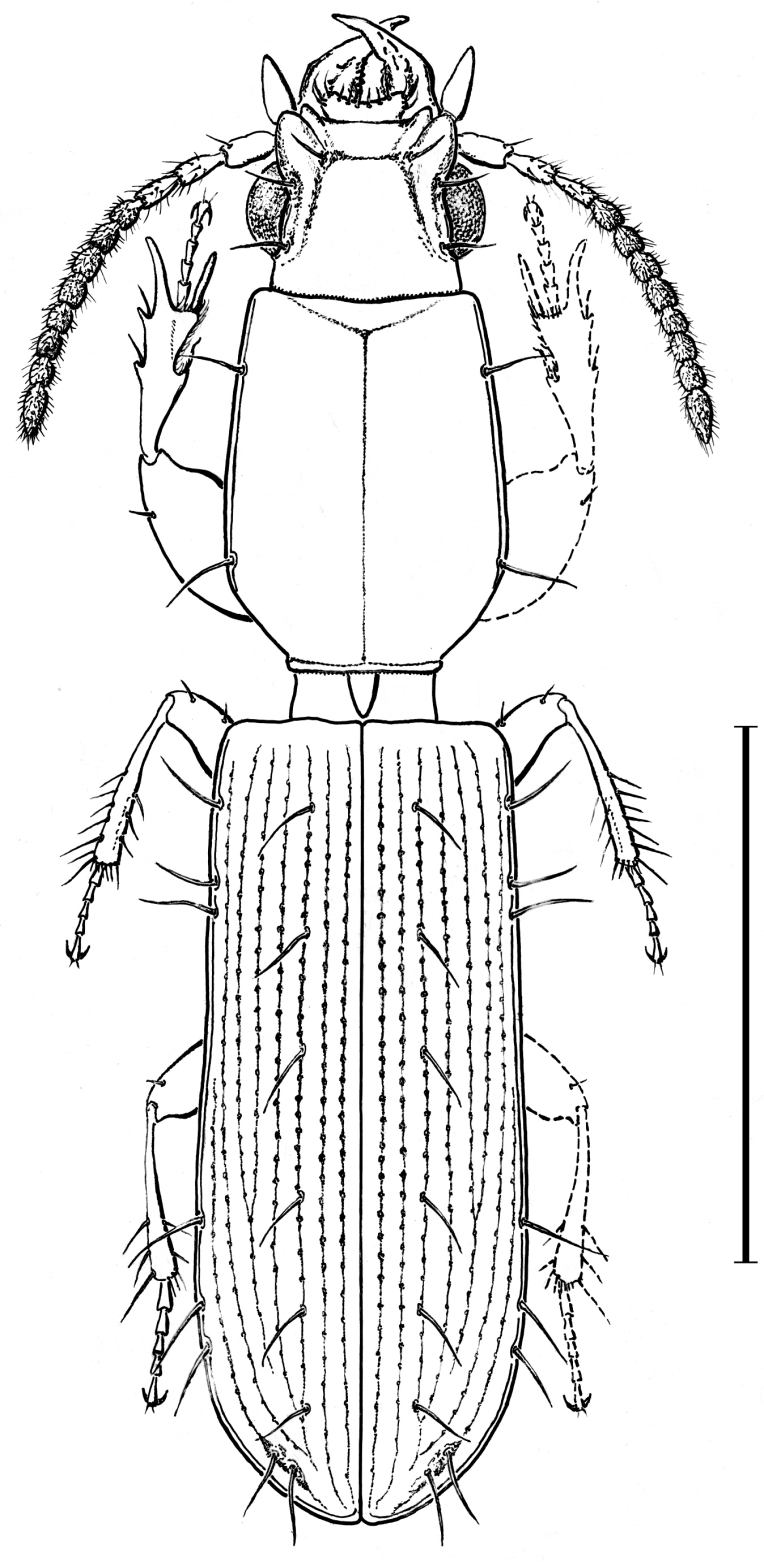
Habitus of *Torretassoa alfierii* Schatzm. & Koch from Iraq. Scale bar 1 mm.

1 specimen: Iraq: Tharthar Lake, vii-viii.1977, leg. J. Pawłowski, on banks of lake, in detritus, together with *D. clypeatus*, (ISEA) – Fig. 1., the first record in Iraq.

##### Comment.

The genus includes a single species described from Heluan in Egypt and then reported from Karaman Island in Yemen (Fedorenko 1996), Iran and Saudi Arabia (Gueorguiev 2011).

#### Genus Clivinopsis

Taxon classificationAnimaliaColeopteraCarabidae

Bedel, 1895

##### Note.

The genus is recognized as including one rare species from North Africa (with two subspecies) and the other one from the Middle Asia.

#### Clivinopsis strigifrons

Taxon classificationAnimaliaColeopteraCarabidae

(Fairmaire, 1874)

##### Studied material.

*Clivinopsis strigifrons*: 1 specimen: Tunisie, Tozeur, iv.1954, R. Demoflys; (PBPC); *Clivinopsis conicicollis* (Reitter, 1909): 1 specimen: Turkmenistan, Mary, Badchyz NSG, Eroyulenduz, 19–20.iv.1993, Cate & Dostal leg., (PBPC).

##### New record.

1 specimen: Iraq: Hatra, 3.v.1978, leg. Z. Stebnicka, (ISEA).

##### Comments.

The locality in Iraq is subequally distant from those of *C. conicicollis* (Turkmenistan, Kazakhstan) and *C. strigifrons* (Algeria, Tunis, Mauretania). We have found no significant difference between all examined specimens of these two species. Unlike of Fedorenko (1996) and Balkenohl (2003) who treated both taxa separately we follow Müller (1937: 130) in recognizing these taxa as conspecific. The validity of *C. strigifrons bonifacei* Bruneau de Miré, 1952 (Mauretania) is unclear.

### Key to Iraqi Dyschiriini species

(including possible species in square brackets)

**Table:** 

1	Head tumid; body cylindrical, redish to testaceous; pronotum distinctly longer than broad	**2**
–	Head not tumid. Other combination of characters.	**3**
2(1)	Head and pronotum anteriorly coarsely rugose. B1, D3, A1, H1. L. about 5 mm	***Clivinopsis strigifrons* (Fairmaire, 1874)** [= *C. conicicollis* Reitter, 1909]
–	Head and pronotum smooth. B0, D5-7, A2, H3. L. about 3 mm	***Torretassoa alfierii* Schatzmayr & Koch, 1933** (Fig. 1)
3(1')	Clypeus tridentate; elytra with basal ridge, fore tibia strongly dentate. B1, D3, A2, H2. L. 3.0 – 4.2 mm	***Dyschirius beludscha ganglbaueri* Znojko, 1927**
–	Clypeus not tridentate, at most broadly vaulted in middle; elytra without, rarely with gently basal ridge, fore tibia less strongly dentate	**Genus *Dyschiriodes* Jeannel, 1941**...**4**
4(3')	Fore tibia with indistinct lateral teeth and with distinctly curved apical spine. B1, D2-3, A2, H1. L. mostly over 4 mm	**5**
–	Fore tibia with distinct marginal teeth (at least lower one)	**6**
5(4)	Pronotum and elytra broader, elytral striae finer. D2. L. 4.0–5.4 mm.	***D. agnatus* (Motschulsky, 1844)**
–	Pronotum and elytra narrower, elytral striae coarser. D3. L. 3.9–4.3 mm	***D. jedlickai* (Kult, 1940)**
6(4')	Basal SP present	**7**
–	Basal SP missing	**10**
7(6)	Elytral striae not weakened apically; lateral channel of pronotum not shortened. D3, H3, A2	**8**
–	Elytral striae weakened to obliterated apically	**9**
8(7)	Head with frons coarsely rugose; elytral base with small tubercle. L. 2.8–3.7 mm	[***D. chalybeus gibbifrons* (Apfelbeck, 1899)**]
–	Head with straight or obsolete clypeofrontal suture; elytral base with two distinct tubercles. L. 3.5–4.9 mm	***D. salinus striatopunctatus* (Putzeys, 1846)**
9(7')	Lateral channel of pronotum not shortened. D3. L. 2.6–3.3 mm	[***D. schaumi* (Putzeys, 1866)**]
–	Lateral channel of pronotum strongly shortened. D2. L. 3.1–4.2 mm	[***D. syriacus* (Putzeys, 1866)**]
10(6')	Lateral channel of pronotum shortened, not reaching posterior SP; elytra non cylindric.	**11**
–	Lateral channel of pronotum not shortened, at least reaching posterior SP	**13**
11(10)	Clypeofrontal suture V-shaped, elytral striae obliterated apically	**12**
–	Clypeofrontal suture straight; lateral channel of pronotum strongly shortened, disappeared just below anterior SP; elytral striae strongly weakened apically. L. 3.1–4.0 mm	***D. importunus importunus* (Schaum, 1857)**
12(11)	Lateral channel of pronotum strongly shortened, disappeared just below anterior SP. D3, A1-2. L. 2.3–3.1 mm	[***D. luticola luticola* (Chaudoir, 1850)**]
–	Lateral channel of pronotum slightly shortened, disappeared just above posterior SP. D3, A1. L. 2.6–3.0 mm	***D. cariniceps* (ab. kalalae Mařan, 1935)**
13(10')	Elytra cylindric; striae deep apically	**14**
–	Elytra shorter, ovate to elliptic	**17**
14(13)	Each elytron with two distinct, often fused tubercles at base. L. 3.7–5.0 mm	[***D. cylindricus hauseri* (Fleischer, 1898)**]
–	Elytra without or with a small basal tubercle	**15**
15(14)	Larger species. L. 3.9–5.0 mm. Elytral base without tubercles	***D. auriculatus* (Wollaston, 1867)**
–	Smaller species. L. 2.3–3.4 mm. Elytral base with one small tubercle	**16**
16(15)	Clypeofrontal suture prolonged posteriorly by distinct keel; pronotum and elytra in average narrower. L. 2.1–3.3 mm	***D. clypeatus* (Putzeys, 1866)**
–	Clypeofrontal suture not prolonged posteriorly by distinct keel; pronotum and elytra in average broader. L. 2.3–3.4 mm	***D. pusillus* (Dejean, 1825)**
17(13')	Elytral base with two tubercles; striae deep throughout. A2. L. 2.9–3.7 mm	***D. euphraticus* (Putzeys, 1846)**
–	Elytral base with 0-1 tubercles. A1	**18**
18(17)	Elytral base with one tubercle; elytra longer, with striae not to slightly weakened apically. D0-3. L. 2.2–3.2 mm	***D. mesopotamicus* (Müller, 1922)**
–	Elytral base without distinct tubercle; elytra shorter, with striae disappeared to strongly weakened apically. D3. L. 2.5–3.0 mm	***D. cariniceps* (Baudi di Selve, 1864)**

## Supplementary Material

XML Treatment for Clivina

XML Treatment for Clivina (Clivina) ypsilon

XML Treatment for Clivina (Clivina) euphratica

XML Treatment for Clivina (Leucocara) laevifrons

XML Treatment for Dyschirius

XML Treatment for Dyschirius beludscha ganglbaueri

XML Treatment for Dyschiriodes

XML Treatment for Dyschiriodes (Dyschiriodes) agnatus

XML Treatment for Dyschiriodes (Dyschiriodes) auriculatus

XML Treatment for Dyschiriodes (Dyschiriodes) cariniceps

XML Treatment for Dyschiriodes (Dyschiriodes) clypeatus

XML Treatment for Dyschiriodes (Dyschiriodes) euphraticus

XML Treatment for Dyschiriodes (Dyschiriodes) jedlickai

XML Treatment for Dyschiriodes (Dyschiriodes) mesopotamicus

XML Treatment for Dyschiriodes (Dyschiriodes) pusilluspusillus

XML Treatment for Dyschiriodes (Dyschiriodes) salinusstriatopunctatus

XML Treatment for Dyschiriodes (Eudyschirius) importunusimportunus

XML Treatment for Torretassoa alfierii

XML Treatment for Clivinopsis

XML Treatment for Clivinopsis strigifrons
